# Challenges and outcomes of pregnancy in an uncorrected Tetralogy of Fallot with pulmonary atresia and major aorta-pulmonary collateral arteries (MAPCA): a case report

**DOI:** 10.1186/s43044-023-00335-8

**Published:** 2023-02-02

**Authors:** Surekha Thakur, Neha Rawat, Bharti Sharma, Pooja Sikka, Neeti Dogra, Neelam Aggarwal, Vanita Suri, Rajesh Vijayvergiya, Atit A. Gawalkar

**Affiliations:** 1 Department of Obstetrics and Gynaecology, PGIMER Chandigarh, Postgraduate Institute of Medical Education and Research, Chandigarh, 160012 India; 2 Department of Anesthesia, PGIMER Chandigarh, Postgraduate Institute of Medical Education and Research, Chandigarh, 160012 India; 3 Department of Cardiology, PGIMER Chandigarh, Postgraduate Institute of Medical Education and Research, Chandigarh, 160012 India

**Keywords:** Congenital heart disease, Tetralogy of Fallot, Tetralogy of Fallot with major aortopulmonary collateral arteries, Pregnancy

## Abstract

**Background:**

Tetralogy of Fallot is a severe type of congenital heart disease (CHD) and one of the leading indirect causes of mortality & morbidity among women with CHD. We came across a rare case of an uncorrected Tetralogy of Fallot with pulmonary atresia and major aortopulmonary collateral arteries in pregnancy.

**Case presentation:**

We are reporting the challenges in managing a pregnancy of 25-years-old G3 P0110, previous one stillbirth and who was diagnosed to have congenital heart disease during pregnancy following spontaneous abortion.

**Conclusions:**

This case report highlights the role of multidisciplinary care in managing such a high risk case. It also emphasizes the role of cardiac examination of every woman before pregnancy so that definitive treatment or optimization can be done in time for a better outcome.

**Supplementary Information:**

The online version contains supplementary material available at 10.1186/s43044-023-00335-8.

## Background

Tetralogy of Fallot(TOF) is the most prevalent cyanotic congenital heart disease (CHD), characterized by pulmonary stenosis (PS), ventricular septal defect (VSD), overriding aorta and right ventricular hypertrophy (RVH). In place of pulmonary stenosis, pulmonary atresia (PA) is an uncommon variant of TOF, with an estimated frequency of 0.7 per 10,000 live births [1].

In PA, the blood supply to lungs are provided with major aorta-pulmonary collateral arteries (MAPCA), is the most severe type of TOF, accounting for only 20% of TOF [2]. We are reporting an extremely rare case of uncorrected TOF with PA with MAPCA, who had successful pregnancy outcome.

## Case presentation

A 25-year-old woman, G3P1L0A1 presented at 17 weeks of gestation. She was a known case of TOF with PA and the lesions were uncorrected till now.At 22 years of age she conceived spontaneously and had preterm vaginal delivery of a stillborn at local hospital. Then she again conceived after one year and had spontaneous abortion at 8 weeks of gestation. During that time treating physician noticed grade 4 clubbing, cyanosis and low arterial saturation of 85% by oximetry and she was referred to our centre for further evaluation.

**Fig. 1:**
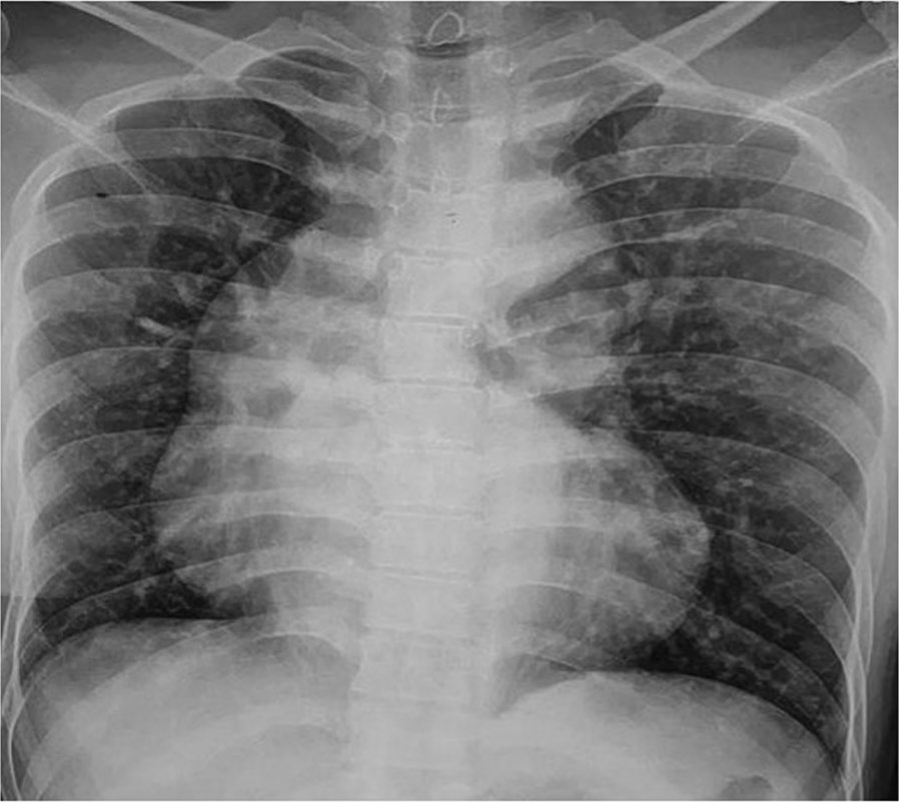
Chest X ray-posteroanterior projection showing boot shaped heart and dilated aorta

**Fig. 2:**
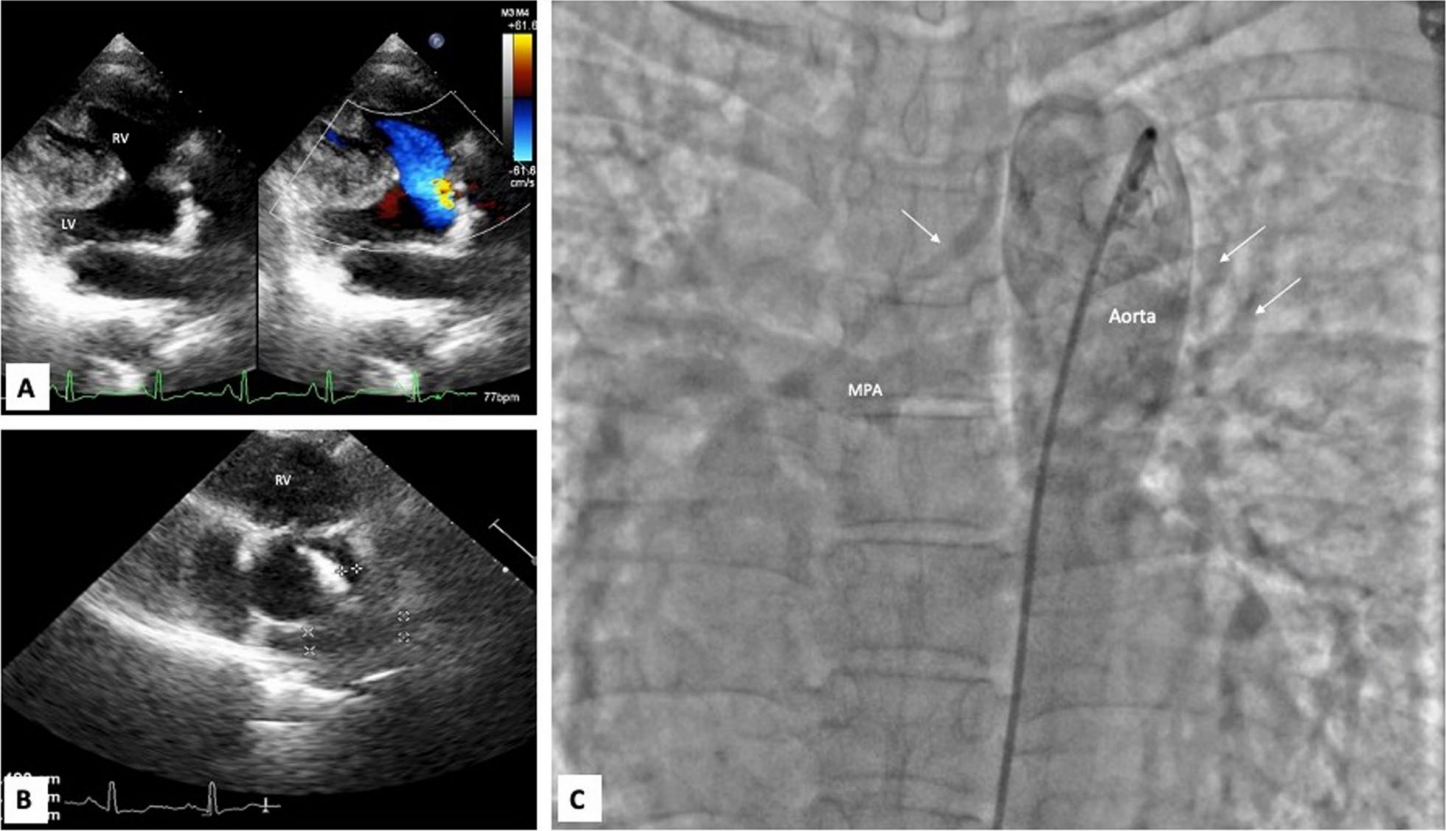
**A** Parasternal long axis view of echocardiography showing large ventricular septal defect with right to left shunt, right ventricular hypertrophy and malaligned aorta. **B** Parasternal short axis view of echocardiography at the level of aortic valve showing pulmonary valve atresia and confluent small pulmonary arteries. **C** Invasive angiogram with pigtail catheter in descending thoracic aorta showing major aortopulmonary collaterals (white arrows) filling the confluent pulmonary arteries

Her chest X-ray showed boot shaped heart with clear lung fields (Fig. 1). Transthoracic echocardiography showed hypertrophic right ventricle, large misaligned subaortic ventricular septal defect with right to left blood flow, more than 50% aortic override, no visible flow across pulmonary valve and small pulmonary arteries (features suggestive of TOF with PA) (Fig. 2A and B) (Additional file 1: Video S1). CT Pulmonary Angiography (CTPA) was also done which showed more than 50% aortic override, main pulmonary artery (MPA) was not visualized, right pulmonary artery measured 12 mm and left pulmonary artery measured 8 mm and prominent MAPCAs were seen. Aortic root measured 4.3 cm,ascending aorta 4.8 cm, arch of aorta (mid)2.8 cm, DTA (proximal) 2.8 cm and DTA (hiatus)1.4 cm. Invasive cardiac catheterization showed both left ventricle and right ventricle emptying completely into aorta, large ventricular septal defect, dilated aortic root and multiple MAPCAs emptying into confluent pulmonary arteries (Fig. 2C) (Additional file 1: Video S2). She was planned for intra cardiac repair (ICR) in follow up and was started on oral propranolol 40 mg once daily.

She however failed to follow up due to the COVID pandemic and presented in her second trimester of index pregnancy. On examination her pulse rate was 82 regular beats per minute, blood pressure was 100/60 mmHg, arterial saturation of 80% by oximetry at room air. She had grade 4 clubbing in fingers and toes. Her body mass index was 18 kg/m^2^ and per abdomen fundal height was corresponding the period of gestation. She had NYHA class 2 dyspnoea on exertion. Further management was discussed by a cardio-obstetric team. She was explained about the maternal and fetal risks, but she and her family decided to continue the pregnancy. On laboratory assessment, hemoglobin was 20.1 g/dl, hematocrit was 65.5% and platelet count was 87,000/mm^3^. Fetal ultrasound and cardiac screening were normal. Therapeutic phlebotomy was performed at 26 weeks of gestation when her hematocrit was 65.5%. Her platelet counts also improved to 120,000/mm^3^ and she was started on oral Ecosprin 75 mg once a day for polycythemia. In the 3^rd^ trimester her haematocrit remained between 53 and 63%. She was on regular follow up in our cardio-obstetric clinic. At 28 weeks she was diagnosed with (fetal growth restriction) (FGR) and was followed up biweekly for fetal surveillance. Doppler studies showed absent end diastolic flow (AEDF) at 33 weeks of pregnancy and at 34 weeks she was admitted for early delivery in view of severe FGR (< 3rd centile) with AEDF. Upon admission, she was hemodynamically stable with SpO2 of 85% at room air, hemoglobin was 19 g/dl, hematocrit was 60% and platelet count was 64,000/mm^3^.

She underwent emergency caesarean for fetal distress under general anaesthesia. Preinduction arterial access and central venous access were established. Modified rapid sequence induction was done with preoxygenation (ETO2- 90) in left up tilt of 30 degrees. Fentanyl 100 mics, titrated dosed of ketamine 80 mg and thiopentone 50 mg weregiven according to hemodynamicsand succinylcholine 100 mg was given for induction. She delivered a live born baby of 1198 g (< 3rd centile), with Apgar score of 6, 8. In post-operative period after 4 h of caesarean she had atonic peripartum hemorrhage (PPH) which was managed with uterotonics and Bakri balloon insertion. Two units of single donor apheresis platelet(SDAP) were transfused as her post operative platelet count was 57,000/mm3and sonoclot showed platelet dysfunction. She remained stable thereafter and was discharged on day 7 of postpartum and advised for further follow up in cardiology OPD. Baby had some feeding issue secondary to AEDF and hyperbilirubinemia, managed and was discharged after 15 days. At 6 weeks of follow up both baby and mother are doing well.

## Conclusions

Uncorrected TOF in adulthood is rare event and more rare in pregnancy, as it usually present within the first year of life with cyanosis, failure to thrive, hypoxic episodes or transient loss of consciousness. The clinical features of TOF in adults are related to the severity of the anatomic defects which can be palpitations, dyspnea on exertion, syncope, heart failure, arrhythmias, and RV dysfunction [3]. Early total corrective surgery is the best treatment option. Majority of untreated patients probably die during childhood, 1% of the untreated patients survive by the age 50 years [4, 5]. In case of pulmonary atresia, development of pulmonary collaterals enables patients to reach into adulthood as seen in our case. TOF with pregnancy is even rarer.

Most of the cases of uncorrected TOF are reported from developing countries, remains an important cause of maternal morbidity and mortality and have significant effects on fetal outcome [6]. Among patients with TOF who conceive, adverse fetal outcome were high in patients with unrepaired TOF compared to repaired TOF [7]. Several other factors portending adverse outcomes are presence of morphologic pulmonary artery abnormalities (ductal origin of PA, hypoplastic or disconnected PA), arterial oxygen saturation < 85% and hemoglobin concentration of > 20 g/dl, higher RV systolic pressure, and younger age at primary surgical repair [7, 8]. Index case falls under WHO Risk Class III, which has significantly increased risk of maternal mortality or severe morbidity and as per CARPREG score, the risk of mortality ranges between 5 and 15% [6]. Index case was also informed regarding the associated maternal morbidity and fetal complications like fetal loss, FGR (fetal growth restriction), congenital heart defects; however she opted to continue her pregnancy.

Management of such patients is challenging due to hemodynamic variations. The physiological changes of pregnancy lead to plasma volume expansion, concomitant fall in both systemic vascular resistance (SVR) and pulmonary vascular resistance (PVR). This change in the delicate balance of SVR and PVR, changes the blood flow across the PDA and MAPCAs. Fall in SVR more than PVR leads to decreased flow of blood across the PDA and MAPCA, this leads to pulmonary oligemia and decreases the oxygenation in lungs. Increase in PVR would also have the same physiological consequence [9]. Coagulopathy is associated with TOF poses another problem during hemostasis and during the postpartum period as seen in index case. The secondary polycythemia due to chronic hypoxia leads to decreased thrombopoesis. Additionally, the platelet aggregation and function are also impaired due to increased PCV.

Maternal complications in these patients are right ventricular failure, worsening of cyanosis and dyspnea, thromboembolism and maternal mortality. Fetal complications include abortions, FGR, prematurity, and fetal loss. Our patient previously had stillbirth, abortion and FGR in index pregnancy. Maternal cyanosis and polycythemia are the main factors leading to FGR due to low oxygen saturation.

Due to scarcity of reported literature, the obstetric management i.e. mode of delivery, uterotonics to be used, type of anesthesia is challenging. In index case patient was given a trail of vaginal delivery with continuous monitoring, oxygen supplementations, labor analgesia and infective endocarditis prophylaxis. However, she landed up to emergency cesarean section for fetal distress. Regarding the choice of utertonics, index case was managed with oxytocin infusion with vigilant monitoring however Gomez et al. had preferred methylergonovine over oxytocin as they found significant reduction in SVR with oxytocin [10].

During general anaesthesia, it is crucial to maintain the pre operative balance between PVR and SVR to avoid any worsening of the pre-existing right to left shunt, therefore in index case pre-induction arterial access was taken for beat to beat monitoring and central venous access for administering vasopressors and vasodilators. To avoid the intubation response patient was given fentanyl 100 mics given before induction and Xylocaine 100 mg was given during rapid sequence induction for blunting of laryngoscopic response.

It is also important to avoid hypoxia, respiratory acidosis and hypothermia during surgery by maintaining adequate oxygenation, controlled ventilation and using warming devices to maintain PVR. Our patient had baseline saturation of 80% on room air, our anaesthetic goal was to maintain spo2 between 80 to 88% Preload was maintained with bolus of ringer lactate during induction 300 ml. Blood loss was minimised with administering oxytocin via infusion pump titrated to hemodynamics and uterine tone. Phenylephrine bolus was given to manage the fall in SVR due to oxytocin.

Careful monitoring is required during delivery and postpartum period due to sudden cardiovascular alteration. Patients who remain stable can achieve successful pregnancy outcome and the mode of delivery can be made on obstetric indications. Multidisciplinary approach is key aspects for management of these cases.

## Supplementary Information

**Additional file 1.** Echocardiography video.

**Additional file 2.** Cath-angiography Video.

## Data Availability

Yes,data supporting the findings of echocardiography and Cath angio has been submitted as supplementary files for reviewers.

## Abbreviations

AEDF: Absent end diastolic flow
CHD: Congenital heart disease
CTPA: CT Pulmonary Angiography
FGR: Fetal growth restriction
ICR: Intra cardiac repair
MAPCA: Major aorta-pulmonary collateral arteries
MPA: Main pulmonary artery
NYHA: New York Heart Association
PA: Pulmonary atresia
PPH: Post partum hemorrhage
PS: Pulmonary stenosis
RVH: Right ventricular hypertrophy
SDAP: Single donor apheresis platelet
TOF: Tetralogy of Fallot
VSD: Ventricular septal defect

## References

[1] O'Leary PW, Edwards, William D, et al. Moss and Adams' Heart Disease in Infants, Children and Adolescents. In: Allen HD, Driscoll DJ, Shaddy RE, Feltes TF, editors. Lippincott Williams & Wilkins, Philadelphia 2008.

[2] Liao PK, Edwards WD, Julsrud PR (1985). Pulmonary blood supply in patients with pulmonary atresia and ventricular septal defect. J Am Coll Cardiol.

[3] Brickner ME, Hillis LD, Lange RA (2000). Congenital heart disease in adults second of two parts. NEJM.

[4] Semeraro O, Scott B, Vermeersch P (2008). Surgical correction of tetralogy of Fallot in a seventy-five year old patient. Int J Cardiol.

[5] Fairley SL, Sands AJ, Wilson CM (2008). Uncorrected tetralogy of Fallot: adult presentation in the 61st year of life. Int J Cardiol.

[6] Siu SC, Sermer M, Colman JM, Alvarez AN, Mercier LA, Morton BC (2001). Prospective multicenter study of pregnancy outcomes in women with heart disease. Circulat.

[7] Veldtman G, Connolly H, Grogan M (2004). Outcomes of pregnancy in women with tetralogy of fallot. J Am Coll Cardiol.

[8] Presbitero P, Somerville J, Stone S, Aruta E, Spiegelhalter D, Rabajoli F (1994). Pregnancy in cyanotic congenital heart disease: outcome of mother and fetus. Circulation.

[9] Warnes CA, Oakley C, Warnes CA (2007). Cyanotic congenital heart disease. Heart disease in pregnancy.

[10] Gomez LM, Jones RC, Fuertes MR, Tate DL, Ramanathan J (2018). Pregnancy with uncorrected tetralogy of Fallot (TOF), pulmonary atresia and major aorto-pulmonary collateral arteries (MAPCA). Case Rep Perinatal Med.

